# Differential Expression of Programmed Cell Death on the Follicular Development in Normal and Miniature Pig Ovary

**DOI:** 10.1371/journal.pone.0046194

**Published:** 2012-10-08

**Authors:** Sang Hwan Kim, Kwan Sik Min, Nam Hyung Kim, Jong Taek Yoon

**Affiliations:** 1 Department of Animal Life Science, Hankyong National University, Ansung, Gyeonggi-do, Korea; 2 Institute of Genetic Engineering, Hankyong National University, Ansung, Gyeonggi-do, Korea; 3 Graduate School of Bio and Information Technology, Hankyong National University, Ansung, Gyeonggi-do, Korea; 4 Department of Animal Sciences, Chungbuk National University, Cheongju, Chungbuk, Korea; Baylor College of Medicine, United States of America

## Abstract

Follicles are important in oocyte maturation. Successful estrous cycle requires remodeling of follicular cells, and proper execution of programmed cell death is crucial for normal follicular development. The objectives of the present study were to understand programmed cell death during follicle development, to analyze the differential follicle development patterns, and to assess the patterns of apoptosis and autophagy expression during follicle development in normal and miniature pigs. Through the analysis of differential patterns of programmed cell death during follicular development in porcine, MAP1LC3A, B and other autophagy-associated genes (ATG5, mTOR, Beclin-1) were found to increase in normal pigs, while it decreased in miniature pigs. However, for the apoptosis-associated genes, progression of genes during follicular development increased in miniature pigs, while it decreased in normal pigs. Thus, results show that normal and miniature pigs showed distinct patterns of follicular remodeling manifesting that programmed cell death largely depends on the types of pathway during follicular development (Type II or autophagy for normal pigs and Type I or apoptosis for miniature pigs).

## Introduction

The miniature pig is an important animal model in biomedical research, including investigations of cardiovascular dysfunction, gastric function, oncology, and tissue transplantation. However, compared with the common domestic pig, less information regarding the production of cloned and genetically modified pigs is available for the miniature pig [1].

In addition, normal pig follicle development differs significantly from that in miniature pigs [2]. In all mammals, ovarian follicle development, ovulation, and corpus luteum formation are complex processes accompanied by dramatic changes in follicular cells under specific and strict regulation by steroid hormones and growth factors [3]. Many investigators have studied which factors directly or indirectly regulate and modulate the ovulation rate, and how many follicles grow or die during atresia [4]–[6].

Developing follicles play an important role in oocyte development. There are 4 basic stages in ovarian follicle development: primordial, primary, secondary, and tertiary or Graafian follicles [5], [7].

The pig follicles growth and development probably takes place continuously from days 13 to 14 of the estrous cycle to ovulation [8], [9]. And after day 16 of the estrus cycle, approximately 160 to 200 tertiary follicles are present, and then 150 to 190 follicles degenerate and disappear from the ovaries through the process of atresia [10], [11].

Therefore, during follicular growth and development in pigs, more than 99% of follicles selectively disappear [6], [12].

Recently, both autophagy and apoptosis of mouse ovary cells were found to occur upon nutrient depletion towards the end of batch culture [13]. Furthermore, autophagy and apoptosis gene engineering is considered to play an important role in mammalian ovary cell development [14].

Cells have 2 major programmed cell death systems. One is the apoptotic cell degradation system, which is responsible for the selective degradation of most short-lived proteins (type I pathway) [15], [16]. The other is the lysosomal system wherein proteins from both inside and outside of the cell are delivered to the lytic compartment (type II pathway). While non-lysosomal cell death has not been commonly observed, apoptosis and autophagic programmed cell death are prominent during the development of animals from diverse taxa [17].

Therefore, this study was conducted to understand the programmed cell death during follicular development, to analyze differential follicle development patterns, and to assess the patterns of autophagy and apoptosis–associated gene expression in the follicle development stage on day 15 of estrous cycle of normal and miniature pig ovaries.

## Materials and Methods

### Ovarian tissues of normal and miniature pigs

Ovarian tissues of normal pigs, on day 15 of their estrous cycle, were collected from a local slaughterhouse at Pyeong-Nong, Pyeongtaek, Korea. The samples were placed into an LN2 freezer box and were transported to the laboratory within 2 hours. The ovarian tissues of miniature pigs, on day 15 of estrous cycle (luteal phase), were obtained via laparotomy under general anesthesia from Medi Kinetics Co., Ltd. (Pyeongtaek, Korea). This study was carried out in strict accordance with the recommendations in the Guide for the Care and Use of Laboratory Animals of the National Institutes of Health. The protocol was approved by the Committee on the Ethics of Animal Experiments of the Hankyong National University (Permit Number: 2012-1). All surgery was performed under sodium pentobarbital anesthesia, and all efforts were made to minimize suffering.

### Expression of autophagy and apoptosis genes

Total RNA was extracted from porcine ovarian tissue scrapes (corpus luteum was removed) using TRIzol reagent (Invitrogen) and Dnase treated (Ambixon,TX,USA). The extracted RNA was quantified using UV spectrophometry. First-strand cDNA synthesis was achieved by reverse transcription of mRNA (1.0 µg) using an Oligo (dT) primer and SuperScripttm II Reverse Transcriptase (Invitrogen, Grand Island, NY). Afterwards, 1 µL of the cDNA synthesis reaction was added to a SYBR Green (TOYOBO, Tokyo, JPN) master mixture, and PCR amplification was performed using target gene primers (Table 1) with an annealing temperature of 60–65°C for 30 cycles. Report generated analysis was performed using Rotor-Gene Real-Time Software 6.0. Finally, the relative gene expression was analyzed using the 2−ΔΔC_t_ method by normalization to porcine glyceraldehyde-3-phosphate dehydrogenase (GAPDH) mRNA levels.

**Table 1:** Primers for real time PCR analysis of hormone receptors, autophagy and apoptosis-associated genes.

Primer name	Sequence	Product size (base pair)
Porcine GAPDH Fw	*5′ CCCGTTCGACAGACAGCCGTG 3′*	*238*
Porcine GAPDH Rv	*5′ CCGCCTTGACTGTGCCGTGG 3′*	
Porcine MAP1LC3A Fw	*5′ AGAAGCAGCTGCCAGTCCTGGACA 3′*	*687*
Porcine MAP1LC3A Rv	*5′ CAGGCAGGCCTGAGCAATCTTTATT 3′*	
Porcine ATG5 Fw	*5′ AGAGAAGTCTGTCCTTCCGCAGTCG 3′*	*241*
Porcine ATG5 Rv	*5′ AAGCAGAAGGGTGACATGCTCTGGT 3′*	
Porcine mTOR Fw	*5′ CTTTGTCCAGACCATGCAGCAGC 3′*	*120*
Porcine mTOR Rv	*5′ TCGTTGATGCCCTGTAGGTTCAGCT 3′*	
Porcine Beclin-1 Fw	*5′ TGGCGGAAAATCTCGAGAAGGTCCA 3′*	*230*
Porcine Beclin-1 Rv	*5′ TGTGCCAAATTGTCCACTGTGCCAA 3′*	
Porcine Casp-3 Fw	*5′ CATGGTCAGGCCTTGTGAAGCTGAC 3′*	*150*
Porcine Casp-3 Rv	*5′ TCTTCTTCATGACCTCACCGTCGGG 3′*	
Porcine 20α-HSD Fw	*5′ GCCATTGCCAAAAAGCACAAG 3′*	*210*
Porcine 20α-HSD Rv	*5′ GGAAAGCGGATAGTCAGGGTGATC 3′*	
Porcine FSH-r Fw	*5′ GGTGTCACTAGAGGAGGACA 3′*	*430*
Porcine FSH-r Rv	*5′ CAAAACCCAATACCACAACT 3′*	
Porcine LH-r Fw	*5′ CAGTGAAAAAGCCAGCAACA 3′*	*427*
Porcine LH-r Rv	*5′ GAAAGCACAGCAAGGAGACC 3′*	
Porcine VEGFa Fw	*5′ TCACCAAGGCCAGCACATAGGAGA 3′*	*164*
Porcine VEGFa Rv	*5′ TGCAGGAACATTTACACGTCTGCG 3′*	
Porcine IGF-1 Fw	*5′ CAAATGTACTTCCTTCTGAG 3′*	*331*
Porcine IGF-1 Rv	*5′ CTCTTCGCATCTCTTCTAC 3′*	
Porcine PAPP-A Fw	*5′ AACATCTGGATGACCTTC 3′*	*364*
Porcine PAPP-A Rv	*5′ CAACACTCCTTACAACAACT 3′*	

### *In situ* hybridization of MAP1LC3A mRNA

Digoxigenin-labeled antisense and sense complementary MAP1LC3A RNA probes were prepared as previously described. Using recommended protocols, *in-situ* hybridization was performed using the Digoxigenin-labeled hybridization kit (Roshe, Mannheim, GER). For hybridization, ovarian tissues were sliced into 10 µm thick sections. Digoxigenin-labeled probes (200 ng/ml) were hybridized to the ovarian tissue sections using RiboHybe (TOYOBO, Osaka, JPN) hybridization solution at 65°C for 16 hr. Sections were washed in 2× SSC for 5 min at 37°C, and were fixed with 60% formamide in 0.2× SSC three times for 5 min at 37°C. After fixation, the sections were washed in 2× SSC for 5 min at 37°C. The probes were detected with an anti-Digoxigenin antibody (1∶200) in blocking solution and NBT/BCIP stock solution (0.18 mg/ml BCIP, 0.34 mg/ml NBT, and 240 µg/ml levamisole). The samples were incubated for 16 h at room temperature. The slides were briefly dipped in fresh Xylene, dropped with Permount (Fisher, PA, USA) and covered with coverslip.

### Extraction of total protein from the ovary of normal and miniature pigs

For western blot and ELISA, total protein was extracted from ovarian tissues using Pro-prep solution (Intron, Seoul, Korea) according to the manufacturer's instruction. Total protein was quantified using Bradford protein assay (Bio-Rad, CA, USA), and the final protein samples were stored at −80°C.

### Western blot analysis

Each sample containing 30 µg of protein was separated by SDS-PAGE(in duplicate) on a 13% SDS-polyacrylamide gel, and transferred to an Immuno-Blot PVDF membrane (Bio-Rad, CA, USA). The membrane was blocked using blocking buffer (5% non-fat dry milk) overnight at 4°C. Afterwards, the membrane was washed once for 10 min with washing buffer (0.1% Tween 20, 50 mM Tris-HCl (pH 7.6), 200 mM NaCl). The membrane was incubated for 2 hr with anti-rabbit MAP1LC3A, B (Abcam, MA, USA), anti-rabbit ATG5 (Abfrontier, Seoul, Korea), anti-rabbit mTOR (Abfrontier, Seoul, Korea), anti-rabbit Casp-3 (Abcam, MA, USA), anti-rabbit 20α-HSD (Hankyong National University, Ansung, KOR) and anti-rabbit β-actin(Santa Cruz, CA, USA). After binding, the membranes were washed 3 times with 1× TBS-T buffer for 15 min each, and then incubated for 2 hr with HRP-conjugated anti-rabbit and anti-mouse secondary antibodies. The detection was carried out using ECL detection kit with a 5 min incubation in a dark room. The detection reagent was drained and the membrane was exposed to a sheet of diagnostic film in a film cassette for 1 to 30 min.

### Hormone enzyme linked immunosorbent assay

For ELISA, protein samples were diluted in 100 percent assay buffer. Hormones (FSH-receptor, LH-receptor, and 20α-HSD) levels were measured using a quantitative sandwich ELISA (R&D Systems Europe, Abingdon, UK) according to the manufacturer's instruction. All samples were measured in duplicate, and the mean levels were calculated for data analysis. The levels of hormones were determined according to a standard curve, which takes into account 4 parameters based on the following equation: 4 parameters (y = (A−D)/(1+(x/C) ∧ B)+D). The standard curve was calculated from 7 known values. All hormones average fold values were measured as (mean±S.D).

### Immunohistochemistry of apoptosis and autophagy proteins in the ovary of normal and miniature pigs

Immunohistochemistry of apoptosis and autophagy proteins was performed on 5 µm tissue sections mounted on silanized slides. Briefly, paraffin sections were dewaxed with a xylene substitute (Polysciences, PA, USA) and rehydrated in a graded series of ethanol. Antigen retrieval was performed by heating at 95°C in 10 mM sodium citrate (pH 6.0). Endogenous peroxidases were quenched with 0.3% hydrogen peroxide in methanol for 5 min at room temperature. After 3 washes in 1× PBS buffer, the slides were blocked in 1% goat serum containing 3% horse serum for 1 hr at room temperature. Sections were labeled overnight at 4°C with anti-rabbit MAP1LC3A, anti-rabbit ATG5, anti-rabbit Casp-3, anti-rabbit 20α-HSD and anti-Apoptotic detector (Takara, Osaka, JPN). Washed sections were then incubated with anti-rabbit secondary antibodies and Anti-FITC HRP Conjugate (Takara, Osaka, JPN) (diluted 1∶300) for 1 hr at room temperature, and then rinsed and incubated with ABC detection kit (Vector, CA, USA) for 10 min. Diaminobenzidine (Vector, CA, USA) was used as a substrate for HRP. Sections were counterstained with PAS reagent and Harris hematoxylin containing 4% acetic acid. Tissues were dehydrated, cleared, and covered using Permount.

### Fragmentation of isolated chromosomal DNA from the ovary of normal and miniature pigs

The degree of chromosomal DNA fragmentation was quantified as described by Mcconkey et al. (1989). Ovarian tissues of normal and miniature pigs were used to isolate genomic DNA for DNA fragmentation assay. Intact and fragmented chromosomal DNA was prepared from total cells (dead and viable cells), as described previously [18]. Isolated DNA was run on a 1.2% agarose gel. After staining with 1 mg/mL ethidium bromide, the intact and fragmented DNA bands were visualized using an ultraviolet light source.

### Statistical analysis

Data were subjected to a T-test and GLM of the Statistical Analysis System (SAS Institute, version 9.4, Cary, NC, USA). Differences among treatment means were determined by using Duncan's multiple range tests. The statistical significance was established at *p*<0.05.

## Results

### *In-situ* localization of MP1LC3A mRNA in the follicles of normal and miniature pigs

**Figure 1:**
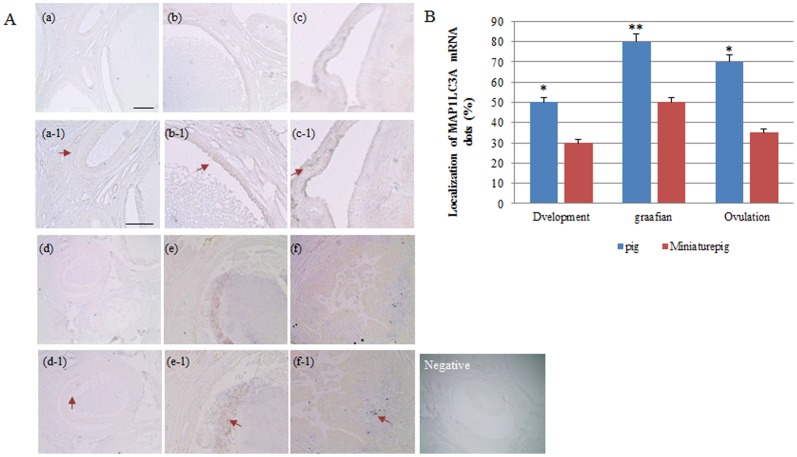
*In situ* hybridization of MAP1LC3A mRNA in the follicular zone of normal and miniature pigs. Prehybridization solution was used as the control for the Graafian follicle in normal pigs below the negative panel. Black bar = 100 um. Red arrows indicate MAP1LC3A RNA probe detection. (A) Ovarian tissue during follicular development in normal and miniature pig ovary. (B) Percentage of MAP1LC3A probe detection dots. ^*,**^Different letters within the same column represent a significant difference (*p*<0.05). **a–c:** Normal pig ovary, **d–f:** Miniature pig ovary, **a, d**) Developing follicle, **b, e**) Graafian follicle, **c, f**) Ovulation stage follicle (a–f: 100× magnification; a-1-f-1: 200× magnification).

Differential MAP1LC3A mRNA expression was detected in normal and miniature pigs during follicle development. MAP1LC3A mRNA expression for normal pig follicles was very high, while for miniature pig follicles was low. Expression was the highest in the granulosa wall of normal and miniature pigs (Figure 1). In this study, expression of MAP1LC3A mRNA in the follicles progressively increased from the developing follicles to the Graafian follicle. According to the results of MAP1LC3A expression during follicles development, the expression of Graafian follicle in normal pig ovary significantly increased (*p*<0.05) among the other follicle stages. The pattern of MAP1LC3A expression in normal pig follicles was different from the miniature pigs.

### Expression of autophagic and apoptotic mRNAs in the ovary tissues of normal and miniature pigs

**Figure 2:**
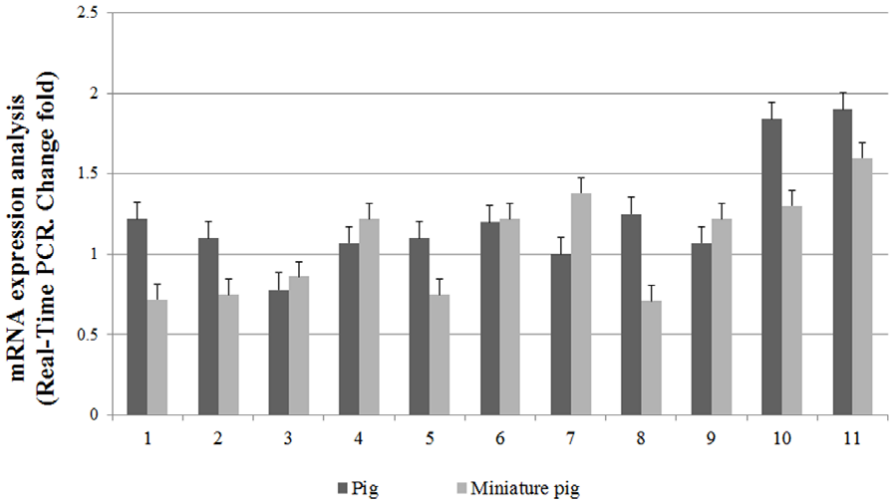
Expression of autophagy and apoptosis-associated gene mRNAs in the ovary of normal and miniature pigs. Experiments were repeated three times, and data are expressed as mean(±S.D). Total RNA was extracted and analyzed by RT-PCR. Lane 1 : porcine-MAP1LC3A, Lane 2 : ATG 5, Lane 3 : mTOR, Lane 4 : Casp-3, Lane 5 : Beclin-1, Lane 6 : 20α-HSD, Lane 7 : FSH-receptor, Lane 8 : LH-receptor, Lane 9 : VEGF, Lane 10 : PAPP-A, Lane 11 : IGF-1.

The results of the analysis showed that the mRNA expression of genes associated with autophagy (MAP1LC3A, ATG5, Beclin-1) was high in normal pig ovarian tissues. However, in the ovaries of miniature pigs, Casp-3, 20α-HSD and mTOR mRNA were highly expressed. Genes associated with growth (Pregnancy-associated plasma protein A (PAPP-A) and Insulin-like growth factor (IGF-1)) were highly expressed in normal pigs. Moreover, vascular endothelial growth factor (Figure 2) and FSH receptor mRNA were highly expressed in miniature pigs. On the other hand, LH receptor mRNA were highly expressed in normal pigs. Therefore, the mRNAs of genes associated with autophagy were more highly expressed in normal pig ovaries than genes associated with apoptosis.

### Expression of hormones in ovary tissue of normal and miniature pigs

**Figure 3:**
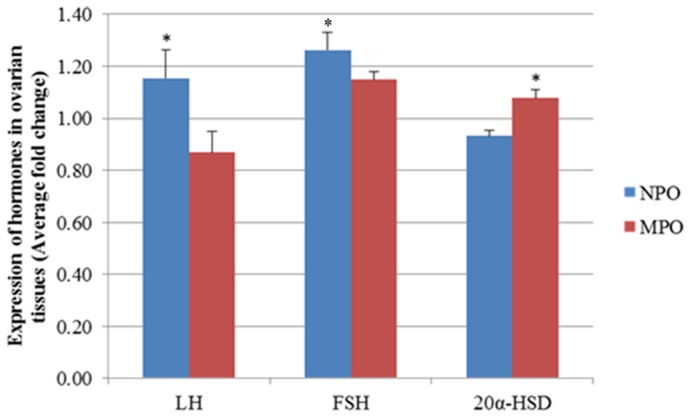
ELISA analysis of FSH, LH, and 20α-HSD proteins in the ovary of normal and miniature pigs. ELISA experiments were repeated three times, and data are average fold change (mean±S.D). *Different letters within the same column represent a significant difference (*p*<0.05). NPO: Normal pig ovary, MPO: Miniature pig ovary.

FSH and LH proteins were strongly detected in the ovary of normal pigs. However, 20α-HSD protein expression was low in the ovary of normal pigs. The pattern of hormone expression in normal pig follicles was apparently different from the pattern of hormone expression in miniature pigs (Figure 3).

### Detection of programmed cell death protein expression in the ovaries of normal and miniature pigs

The protein expression pattern was similar to the mRNA expression pattern.

MAP1LC3A and ATG5 proteins were more highly expressed (p<0.05) in the normal pig ovary than in the miniature pig ovary. And MAP1LC3B protein was highly expressed in normal pig ovaries. However, MAP1LC3B protein expression was not significant different between normal and miniature pig ovaries.

**Figure 4:**
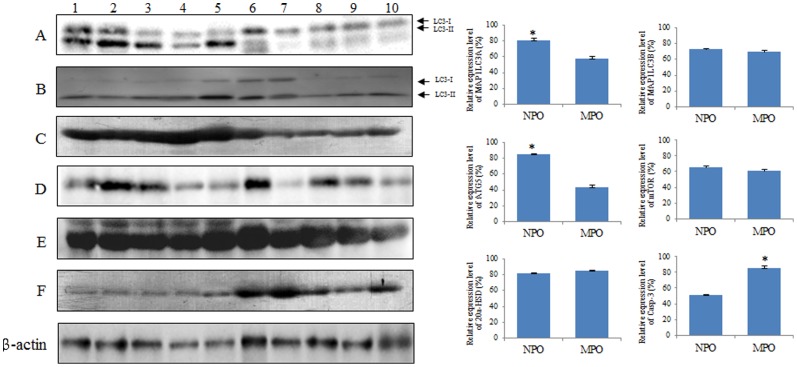
Western blot analysis of autophagy and apoptosis-associated protein in the follicles of normal and miniature pigs. Data represent the mean ± SEM of five individual experiments and were normalized against β-actin (Housekeeping gene) as an internal standard. *Different letters within the same column represent a significant difference (*p*<0.05). Lanes 1–5: Total proteins of normal pig ovary, lanes 6–10: Total proteins of miniature pig ovary. **A**) anti-MAP1LC3A, **B**) anti-MAP1LC3B, **C**) anti-ATG5, **D**) mTOR, **E**) anti-20α-HSD, F) anti-Casp-3.

Results also showed that the 20α-HSD and mTOR proteins expression pattern in normal pigs was within similar level of expression to that of miniature pigs. However, Casp-3 proteins were more highly expressed (*p*<0.05) in the ovary of miniature pigs than in the ovary of normal pigs. In this study, proteins associated with autophagy were more highly expressed in normal pig ovary than proteins associated with apoptosis (Figure 4).

### Immunohistochemistry of autophagic and apoptotic proteins in the follicle stages of normal and miniature pigs

**Figure 5:**
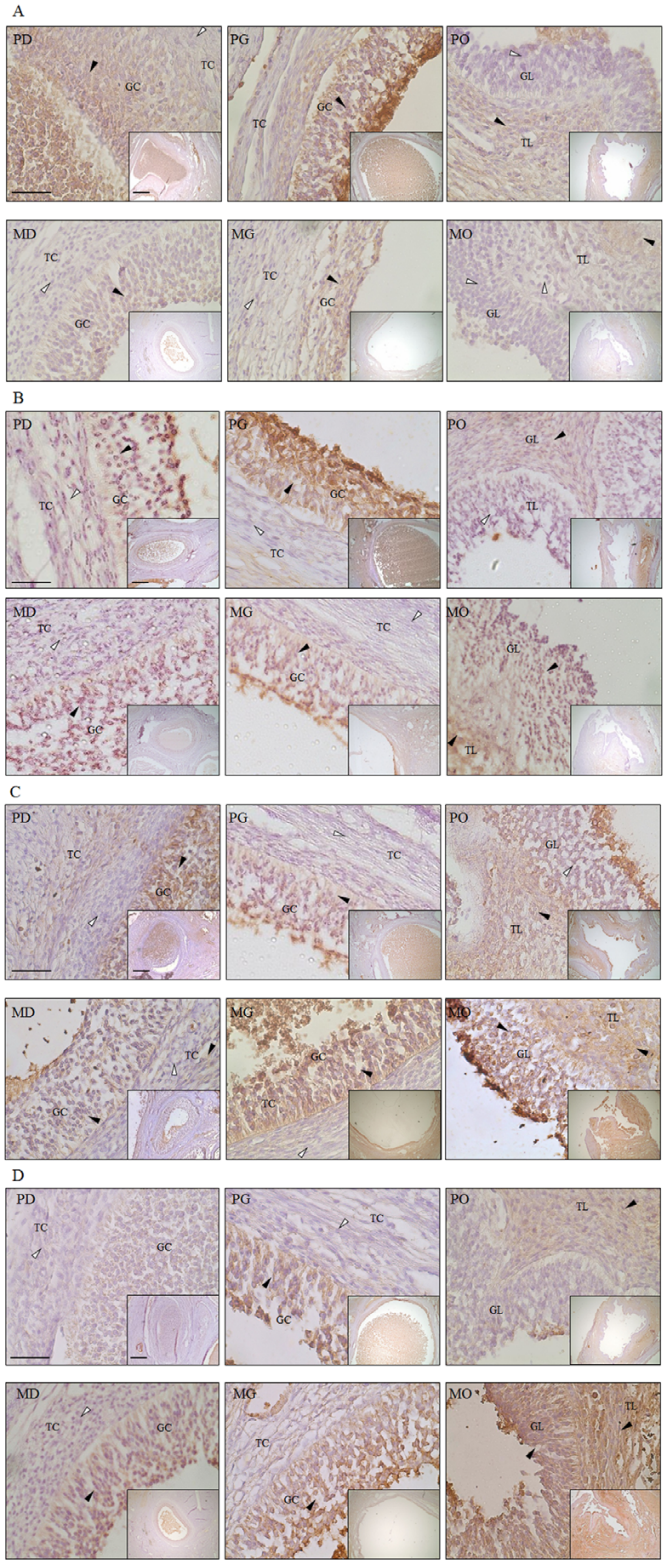
Immunohistochemistry and expression analysis of autophagy and apoptosis-associated proteins in the follicles of normal and miniature pigs. Black arrows indicate protein detection cells, white arrows show non-detection cells. Cells of follicle tissue in normal and miniature pigs were counterstained with hematoxylin. A (large figure) magnification ×400 and A (small figure) magnification ×100. Black bar = 100 um in all figures. **A**) MAP1LC3A, **B**) ATG5, **C**) 20α-HSD, **D**) Casp-3, PD: Normal pig Developing follicle, PG: Normal pig Graafian follicle, PO: Normal pig Ovulation follicle, MD: Miniature pig Developing follicle, MG: Miniature pig Graafian follicle, MO: Miniature pig Ovulation follicle, GC: Granulosa cells, TC: Theca cells, GL: Granulosa lutein cells, TL: Theca lutein cells.

Results of the immunohistochemistry showed that autophagy proteins were highly expressed between the developing and the Graafian pig follicles; however, these proteins were expressed at a low level for miniature pigs follicles. MAP1LC3A and ATG5 proteins were most detected highly in the Graafian follicle of normal pigs. In contrast, these proteins were expressed at a low level in all follicle stages of miniature pigs. However, expression pattern of 20α-HSD proteins in all follicles of normal pigs was within similar level to that of miniature pigs. Additionally, Casp-3 was highly detected in the follicles of miniature pigs. In this study, the expression of autophagy proteins progressively increased during follicular development leading to the Graafian follicle (Figure 5).

### Apoptosis detection and chromosomal DNA fragmentation analysis in the ovary of normal and miniature pigs

**Figure 6:**
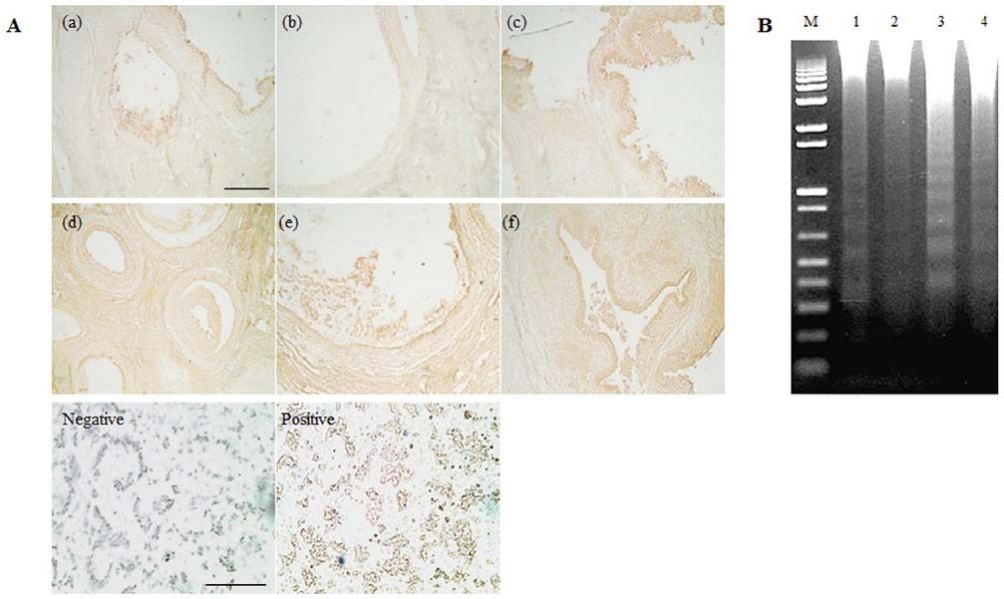
Detection of apoptosis in the follicles of normal and miniature pigs. Apoptosis detection analyses used Terminal deoxynucleotidyl transferase to label 3′-OH ends of DNA fragments that were generated during the process of apoptosis. Detection method used Anti-FITC HRP Conjugate (1∶200) and DAB kit. A (a–f) magnification ×200 and a (negative and positive) magnification ×300. Negative and positive images were analyzed using the follicular cell in cultured for 48 hr. Negative control was without the anti-apoptosis detector. Black bar = 100 um. **A**) Apoptosis detection, **a–c:** Normal pig ovary, **d–f:** Miniature pig ovary, **a, d**) Developing follicle, **b, e**) Graafian follicle, **c, f**) Ovulation stage follicle. **B**) Fragmentation of chromosomal DNA from the ovary of normal and miniature pigs. Equal amounts of chromosomal DNA were electrophoresed on a 1.2% agarose gel. Lane 1–2: Normal pig ovary, Lane 3–4: Miniature pig ovary.

Apoptosis was detected more in miniature pig follicles than in normal pig follicles, and was localized highly in the granulosa wall. To determine whether follicle development in the ovary of normal and miniature pigs was associated with apoptotic cell death, chromosomal fragmentation was examined by agarose gel electrophoresis. Results showed that the follicular cells of miniature pigs underwent remodeling largely through apoptosis, and apoptosis in the follicle of miniature pig increased progressively during follicular development prior to the Ovulation stage follicle (Figure 6).

## Discussion

The objectives of the study include understanding programmed cell death during follicle development in normal and miniature pig ovaries and analyzing differential follicle development patterns in the ovaries of normal and miniature pigs.

The growth and development of the oocyte and its companion somatic cell compartment in the follicle take place in a highly coordinated and mutually dependent manner [19], [20], [21]. In mammalian ovaries, the degeneration of atretic follicles can be explained by the apoptotic cell death of granulosa cells and endocrine cells of the theca internal layer [3], [5], [22], [23]. Therefore, in this study, to assess the patterns of apoptosis and autophagy during follicle development in the ovary of normal and miniature pigs, the differential programmed cell death patterns during follicle development were analyzed.

The miniature pig ovary is known that the number of follicles and the size of the follicles differed from the values reported for standard-sized swine [2], [24]. The expression of MAP1LC3A mRNA in the pig follicles progressively increased during follicular development to the Graafian follicle. In addition, autophagy genes progressively increased in the normal pig ovary. However, autophagy gene expression in the miniature pigs was lower than that in normal pigs.

MAP1LC3A and MAP1LC3B protein were expressed in the majority of normal and miniature pig ovaries. However, significantly higher expression of ovary tissues was found to express the MAP1LC3A protein with strong immunohistochemistry as compared to the miniature pig ovary, suggesting that MAP1LC3A may play a role in normal pig follicular development. This may suggest that MAP1LC3 has expression types with different patterns which plays an important role according to cell and species of animals [25], [26].

FSH and LH receptor proteins were strongly detected in the ovary of normal pigs; however, 20α-HSD protein was expressed at low levels in the ovary of normal pigs. This is consistent with previous reports showing the presence of autophagy [13], [14] and apoptosis [7] during follicle development. These results suggest that the choice of programmed cell death in the follicles is a complex process accompanied by dramatic changes in the follicular cells under the specific and strict regulation of steroid hormones and growth factors [3], [21]. Follicular cell maturation is predominantly the result of increased FSH and LH hormone activation from the primary follicles [5], [7].

In this study, the most highly expressed LH hormone targets in normal pigs were genes associated with autophagy (MAP1LC3A, MAP1LC3B ATG5, Beclin-1), which were progressively activated on the follicles. However, the most highly expressed 20α-HSD targets in miniature pigs were apoptotic family genes (20α-HSD, Casp-3) which were activated on the follicle.

FSH administration induces follicular recruitment and increases the ovulation rate in a dose-dependent manner [27]. The stromal cells form the inner layer of the theca surrounding the developing follicle within the ovary. This vascularized layer of cells responds to luteinizing hormone (LH) by synthesizing and secreting androgens, which are processed into estrogen [28], [29], [30]. Kim et al., (2009) suggested that 20α-HSD played a role in ovary development at least in part by controlling Casp-3 concentrations in bovine ovaries [31].

The immunohistochemistry analysis results showed that programmed cell death (PCD) during follicle development differed between normal and miniature pigs. Autophagy activity in normal pig follicles progressively increased from primary to Graafian. However, it decreased in miniature pigs.

Induction of PCD by different hormones occurs during follicular cell and oocyte development [32]. Hormones are capable of interacting with other cell death gene products that are follicle development factors [33], [34], [35]. An example of such an interaction is between LH and 20α-HSD. When LH and 20a-HSD-expressed factors interact via the inhibition of mTOR and the regulation of Casp-3, programmed cell death is potentiated [30], [31]. Choi et al., (2010) suggested that autophagy involved in determining follicular fate (atresia vs. ovulation) during ovarian follicular development [36].

In conclusion, study of programmed cell death patterns during follicular development of pig ovary showed that follicle development of normal and miniature pigs displayed distinct patterns of follicular remodeling. In addition, results showed that different programmed cell death types were closely related during follicle development in normal and miniature pig ovaries, and that the autophagy had positive effects on the follicular development in pig ovary. Therefore, this study of programmed cell death pathway difference during follicular development in pigs may improve the reproductive efficiency in pigs.

## References

[1] Dor FJ, Tseng YL, Cheng J, Moran K, Sanderson TM, et al (2004) Alpha 1,3-galactosyltransferase gene-knockout miniature swine produce natural cytotoxic antiGal antibodies. Transplantation 78: 15–20. 15257033 10.1097/01.tp.0000130487.68051.eb

[2] Howard PK, Chakraborty PK, Camp JC, Stuart LD, Wildt DE (1982) Correlates of ovary morphology, estrous behavior and eyclicity in an inbred strain of miniature swine. Anat Rec 203: 55–65. 6213180 10.1002/ar.1092030106

[3] Eppig JJ (2001) Oocyte control of ovarian follicular development and function in mammals. Reproduction 122: 829–838. 11732978 10.1530/rep.0.1220829

[4] Byskov AGS (1979) “Atresia.” In: Midgley AR, Sadler WA. (eds) Ovarian follicular development and function. New York; Raven Press; pp. 41–57.

[5] Hirshfield AN (1991) Development of follicles in the mammalian ovary. Int Rev Cytol 124: 43–101. 2001918 10.1016/s0074-7696(08)61524-7

[6] Hughes FM Jr, Gorospe WC (1991) Biochemical identification of apoptosis (programmed cell death) in granulosa cell: evidence for a potential mechanism underlying follicular atresia. Endocrinology 129: 2415–2422. 1935775 10.1210/endo-129-5-2415

[7] McGee EA, Hsueh AJ (2000) Initial and cyclic recruitment of ovary follicles. Endocr Rev 21: 200–214. 10782364 10.1210/edrv.21.2.0394

[8] Clark JR, Brazier SG, Wiginton LM, Stevenson GR, Tribble LF (1982) Time of ovarian follicle selection during the porcine estrous cycle. Theriogenology 18: 697–709.

[9] Dailey RA, Clark JR, Staigmiller RB, First NL, Chapman AB, et al (1976) Growth of new follicles following electrocautery in four genetic groups of swine. J Anim Sci 43: 175–183. 945798 10.2527/jas1976.431175x

[10] Foxcroft GR, Hunter MG (1985) Basic physiology of follicular maturation in the pig. J Reprod Fertil 33: 1–19. 3003359

[11] Guthrie HD, Grimes RW, Cooper BS, Hammond JM (1995) Follicular atresia in pig: measurement and physiology. J Anim Sci 73: 2834–2844. 8582874 10.2527/1995.7392834x

[12] Hsu SY, Hsueh AJW (2000) Tissue-specific Bcl2-protein partners in apoptosis: an ovary paradigm. Physiol Rev 80: 593–614. 10747202 10.1152/physrev.2000.80.2.593

[13] Hwang SO, Lee GM (2008) Autophagy and apoptosis in Chinese hamster ovary cell culture. Autophagy 4: 70–72. 17932458 10.4161/auto.5065

[14] Hwang SO, Lee GM (2008) Nutrient deprivation induces autophagy as well as apoptosis in Chinese hamster ovary cell culture. Biotechnol Bioeng 99: 678–685. 17680685 10.1002/bit.21589

[15] Hershko A, Ciechanover A (1998) The ubiquitin system. Annu Rev Biochem 67: 425–79. 9759494 10.1146/annurev.biochem.67.1.425

[16] Hochstrasser M (1996) Ubiquitin-dependent protein degradation. Annu Rev Genet 30: 405–39. 8982460 10.1146/annurev.genet.30.1.405

[17] Clarke PGH (1990) Developmental cell death: morphological diversity and multiple mechanisms. Anat Embryol 181: 195–213. 2186664 10.1007/BF00174615

[18] Mcconkey DJ, Hartzell P, Jondal M, Orrenius S (1989) Inhibition of DNA fragmentation in thymocytes and isolated thymocyte nuclei by agents that stimulate protein kinase C. J Biologicachle Mistry 264 (23) 13399–13402. 2503500

[19] Matzuk MM, Lamb DJ (2002) Genetic dissection of mammalian fertility pathways. Nat Cell Biol 4: 41–49. 10.1038/ncb-nm-fertilityS41 12479614

[20] Smitz JE, Cortvrindt RG (2002) The earliest stages of folliculogenesis in vitro. Reproduction 123: 185–202. 11866686 10.1530/rep.0.1230185

[21] Thomas FH, Walters KA, Telfer EE (2003) How to make a good oocyte: an update on in-vitro models to study follicle regulation. Hum Reprod Update 9: 541–555. 14714591 10.1093/humupd/dmg042

[22] Tilly JL, Hsueh AJW (1992) Apoptosis as the basis of ovary follicular atresia., In: Hillier SG (eds.). Gonadal Development and Function. New York: Raven Press; pp. 157–165.

[23] Manabe N, Kimura Y, Myoumoto A, Matsushita H, Tajima C, et al. (1998) Role of granulosa cell apoptosis in ovary follicle atresia, In: Yamada T, Hashimoto Y (eds.), Apoptosis: Its Roles and Mechanism. Tokyo: Academic Societies JPNJPN; pp. 97–111.

[24] Day BN (1968) Reproduction in swine, In: Hafez ESE (Ed.) Reproduction in farm animals (2nd Ed.). Philadelphia: Lea and Febiger. pp. 279–288.

[25] He H, Dang Y, Dai F, Guo Z, Wu J, et al (2003) Post-translational modifications of three members of the human MAP1LC3 family and detection of a novel type of modification for MAP1LC3B. J Biol Chem 278: 29278–29287. 12740394 10.1074/jbc.M303800200

[26] Othman EQ, Kaur G, Mutee AF, Muhammad TS, Tan ML (2009) Immunohistochemical expression of MAP1LC3A and MAP1LC3B protein in breast carcinoma tissues. J Clin Lab Anal 23: 249–258. 19623642 10.1002/jcla.20309 PMC6648937

[27] King BF, Britt JH, Esbenshade KL, Flowers WL, Sesti LAC, et al (1993) Ovulatory and endocrine responses after active immunization of gilts against a synthetic fragment of bovine inhibin. J Anim Sci 71: 975–982. 8478297 10.2527/1993.714975x

[28] Gilbert SF (2000) Developmental Biology (6th Edn). Sunderland, MA: Sinauer Associates. pp. 212.

[29] Focchi GR, Simoes Mde J, Baracat EC, de Lima GR, Evencio Neto J (1996) Ultrastructural aspects of the remodeling process of the Corpus albicans in the recent postmenopausal period. Sao Paulo Med J 114: 1173–1176. 9181749 10.1590/s1516-31801996000300006

[30] Sawyer HR (1995) Structural and functional properties of the corpus luteum of pregnancy. J Reprod Fertil 49: 97–110. 7623352

[31] Kim SH, Sin YS, Lee HJ, Chung YH, Min KS, et al (2009) Studies on apoptosis mechanism and 20alpha-Hydroxysteroid Dehydrogenase in the early pregnancy bovine tissues. Reprod Dev Biol 33: 156 Available: http://www.ksar.or.kr/etc/abstract/2009_fall/156.pdf.

[32] Wartenberg H, Ihmer A, Schwarz S, Miething A, Viebahn C (2001) Mitotic arrest of female germ cells during prenatal oogenesis. A colcemid-like, non-apoptotic cell death. Anat Embryol (Berl) 204: 421–435. 11789990 10.1007/s00429-001-0216-7

[33] Billig H, Furuta I, Hsueh AJW (1993) Estrogens inhibit and androgens enhance ovary granulosa cell apoptosis. Endocrinology 133: 2204–2212. 8404672 10.1210/endo.133.5.8404672

[34] Billig H, Furuta I, Hsueh AJW (1994) Gonadotropin releasing hormone directly induces apoptotic cell death in the rat ovary: biochemical and in situ detection of deoxyribonucleic acid fragmentation in granulosa cells. Endocrinology 134: 245–251. 8275940 10.1210/endo.134.1.8275940

[35] Billig H, Chun SY, Eisenhauer K, Hsueh AJW (1996) Gonadal cell apoptosis: hormone-regulated cell demise. Human Reprod 2: 103–117. 10.1093/humupd/2.2.103 9079407

[36] Choi JY, Jo MW, Lee EY, Yoon BK, Choi DS (2010) The role of autophagy in follicular development and atresia in rat granulosa cells. Fertillity and Sterility 93: 2532–2537. 10.1016/j.fertnstert.2009.11.021 20149359

